# Concordance between gene expression in peripheral whole blood and colonic tissue in children with inflammatory bowel disease

**DOI:** 10.1371/journal.pone.0222952

**Published:** 2019-10-16

**Authors:** Nathan P. Palmer, Jocelyn A. Silvester, Jessica J. Lee, Andrew L. Beam, Inbar Fried, Vladimir I. Valtchinov, Fedik Rahimov, Sek Won Kong, Saum Ghodoussipour, Helen C. Hood, Athos Bousvaros, Richard J. Grand, Louis M. Kunkel, Isaac S. Kohane

**Affiliations:** 1 Department of Biomedical Informatics, Harvard Medical School, Boston, Massachusetts, United States of America; 2 Division of Gastroenterology and Nutrition, Boston Children’s Hospital, Harvard Medical School, Boston, Massachusetts, United States of America; 3 Center for Evidence Based Imaging, Brigham and Women’s Hospital, Harvard Medical School, Massachusetts, United States of America; 4 Division of Genetics and Genomics, Boston Children’s Hospital, Departments of Genetics and Pediatrics, Harvard Medical School, Boston, Massachusetts, United States of America; 5 Computational Health Informatics Program, Boston Children’s Hospital, Harvard Medical School, Boston, Massachusetts, United States of America; McMaster University, CANADA

## Abstract

**Background:**

Presenting features of inflammatory bowel disease (IBD) are non-specific. We hypothesized that mRNA profiles could (1) identify genes and pathways involved in disease pathogenesis; (2) identify a molecular signature that differentiates IBD from other conditions; (3) provide insight into systemic and colon-specific dysregulation through study of the concordance of the gene expression.

**Methods:**

Children (8–18 years) were prospectively recruited at the time of diagnostic colonoscopy for possible IBD. We used transcriptome-wide mRNA profiling to study gene expression in colon biopsies and paired whole blood samples. Using blood mRNA measurements, we fit a regression model for disease state prediction that was validated in an independent test set of adult subjects (GSE3365).

**Results:**

Ninety-eight children were recruited [39 Crohn’s disease, 18 ulcerative colitis, 2 IBDU, 39 non-IBD]. There were 1,118 significantly differentially (IBD vs non-IBD) expressed genes in colon tissue, and 880 in blood. The direction of relative change in expression was concordant for 106/112 genes differentially expressed in both tissue types. The regression model from the blood mRNA measurements distinguished IBD vs non-IBD disease status in the independent test set with 80% accuracy using only 6 genes. The overlap of 5 immune and metabolic pathways in the two tissue types was significant (p<0.001).

**Conclusions:**

Blood and colon tissue from patients with IBD share a common transcriptional profile dominated by immune and metabolic pathways. Our results suggest that peripheral blood expression levels of as few as 6 genes (*IL7R*, *UBB*, *TXNIP*, *S100A8*, *ALAS2*, and *SLC2A3*) may distinguish patients with IBD from non-IBD.

## Introduction

Crohn’s disease (CD) and ulcerative colitis (UC) are chronic inflammatory disorders with presenting symptoms similar to those of other intestinal conditions–altered bowel habit, abdominal pain, gastrointestinal bleeding, anorexia and weight loss. Thus, diagnosis of inflammatory bowel disease (IBD) is often delayed, which may have deleterious consequences, particularly for children in whom malnutrition may retard growth[1]. Currently, diagnosis of IBD is based upon a compatible constellation of symptoms, laboratory blood and serum tests, imaging studies, and tissue biopsy, as well as a high index of clinical suspicion[2,3]. Various blood markers have been proposed as potential diagnostic surrogates, including markers of inflammation (e.g., ESR, CRP) and of immunity (ANCA, ASCA and OmpC), but the sensitivity and specificity of these tests are suboptimal[4]. Thus, many children who undergo invasive endoscopic procedures for a clinical suspicion of IBD do not have evidence of CD or UC.

Current hypotheses of IBD pathogenesis implicate genetic predisposition, dysregulated immune responses, environmental factors, and an altered microbiome[5]. As genome-wide analyses has become available, the polygenic nature of IBD has been confirmed in genome-wide association studies (GWAS)[6]. In contrast to GWAS studies, gene expression profiling provides insight into the in situ activity of cellular pathways affected. Several studies have investigated differential gene expression in intestinal biopsies from adults with CD, UC, and non-IBD patients using microarray profiling[7–10]. Most of these studies included patients with long-standing disease who had been exposed to a variety of therapeutic agents. Furthermore, these studies have primarily focused upon gene expression in the actively inflamed tissue, although a few have examined gene expression in peripheral blood[11–16].

We studied a cohort of pediatric patients with incipient IBD because they are treatment-naïve with fewer co-morbidities and lifestyle influences than commonly present in adults. The purpose of our study was to use gene expression analysis to identify a molecular signature of IBD in blood and in colon biopsies and to elucidate key gene pathways involved in disease pathogenesis. The concordance (or lack of it) of gene expression pathways across colon and blood expression in this treatment-naïve pediatric population would also inform us whether the dysregulated pathways were tissue-specific or systemically expressed. A subsidiary aim was to determine the extent to which gene expression in peripheral blood may differentiate children with untreated IBD from similarly symptomatic controls.

## Materials and methods

### Study population and acquisition of intestinal biopsies

Children aged 5–18 years undergoing diagnostic colonoscopy because the treating provider strongly suspected IBD were recruited prospectively at Boston Children’s Hospital from September 2008 to July 2010. A parent/guardian provided written informed consent and minors assented to study procedures. The study protocol was approved by the Boston Children’s Hospital Committee on Clinical Investigation (CCI). Children with a prior diagnosis of IBD or another autoimmune disorder and those receiving corticosteroids, immune modulators or biologics were excluded. Demographic and clinical characteristics recorded on the day of diagnostic colonoscopy were used to calculate Pediatric Crohn’s Disease Activity Index (PCDAI) scores[17] and Pediatric Ulcerative Colitis Activity Index (PUCAI) scores[18] for patients with CD and UC, respectively. Higher scores on these clinician-completed disease activity indices reflect greater symptoms (PCDAI 0 to 100; PUCAI 0 to 85).

Given conflicting data regarding a relationship between colonic biopsy location and gene expression[7,9], and to minimize potential bias, biopsy samples were obtained from the ascending colon or cecum. This is the most commonly involved colonic site for CD, and the plurality of pediatric-onset UC patients present with extensive colitis[19]. Paired endoscopic pinch biopsies were obtained from the most grossly affected area or randomly in patients with a normal appearing right colon. One biopsy was used for histologic examination by the Boston Children’s Hospital Department of Pathology, and the other was immediately stabilized in RNAlater (Ambion, Inc) until RNA was extracted. Peripheral blood was drawn from an indwelling catheter at the time of endoscopy and RNA was extracted using the PAXgene Blood RNA system (PreAnalytiX, Hombrechtikon, Switzerland). Biotinylated cDNA libraries were hybridized to Affymetrix Human Gene 1.0 ST arrays (Affymetrix, Santa Clara, CA).

The diagnosis of IBD was established utilizing clinical, radiographic, and endoscopic findings based upon the Porto criteria[2]. The NASPGHAN algorithm was used to distinguish CD from UC[3]. Those who were found to have a condition other than IBD (e.g., infectious colitis) or normal ileo-colonic histology were designated as symptomatic controls.

### Data analysis

#### Preprocessing and normalization

The Affymetrix Power Tools software package was used to generate probe set (transcript cluster) level measurements using the analysis workflow specification

“-a rma-bg,quant-norm.sketch = 0.usepm = true.bioc = true,pm-only,med-polish” to the apt-probeset-summarize program, corresponding to RMA background correction of the perfect match probes, followed by quantile normalization and median polish summarization. This procedure, and each subsequent step in the analysis, was carried out independently for the colon and blood samples (i.e., all colon samples were processed as a single batch, and all blood samples were processed as a single batch).

Affymetrix transcript identifiers were mapped to Entrez genes using the Bioconductor toolkit[20]. Where multiple transcripts mapped to the same gene, the mean expression intensity across all clusters was computed as a representative value for the gene. All subsequent analyses were performed on these gene-level expression values in the R programming environment[21] unless otherwise specified.

### Statistical analysis

Principal components analysis (PCA) via singular value decomposition (SVD) was performed on a matrix of centered gene-level expression intensities for each sample. Gene-by-gene differential (between the disease groups) expression analysis was performed using a two-tailed Student’s t-test with Bonferroni-adjustment for multiple comparisons and a corrected p< 0.05 was considered significant. The ConsensusPathDB tool[22] was used to map genes differentially expressed between the disease states to Gene Ontology (GO) Biological Process terms (levels 2, 3, and 4)[23], and to the KEGG[24] and Reactome[25] databases with reference to the background genes represented on the microarray and significance of overlap was tested using a hypergeometric distribution.

### Disease state analysis

Using the glmnet package, we fitted a 5-fold cross-validated Lasso (least absolute shrinkage and selection operator) regularized regression model to our pediatric blood mRNA measurements to predict IBD status. The utility of the derived model was tested on an independent set of mRNA measurements from IBD-diagnosed and healthy adults available in the NCBI’s Gene Expression Omnibus (accession GSE3365)[12]. Because the two data sets were measured on different microarray platforms (Affymetrix GeneChip Human Gene 1.0 ST for the present study, Affymetrix Human Genome 133A for the test data set), the absolute mRNA quantitation values after within-dataset normalizations were not directly comparable and required further adjustment to ensure that the coefficients of a model learned on one data set would be applicable to the other. The expression intensities for each sample in both data sets were log transformed, zero-centered, and scaled to unit variance to achieve a notionally common scale, and finally exponentiated to preserve effect size for this analysis. Information about gene function was obtained through reviewing articles retrieved using the HGNC gene name as a search term in PubMED.

## Results and discussion

Useable peripheral blood samples were obtained from 98 subjects (78 of whom also had available biopsy samples; Table 1). The median age at the time of diagnostic colonoscopy was 14.2 years (IQR 11.3–16.2). There was one subject with CD whose biopsy sample was obtained from the sigmoid colon as the colonoscopy was terminated prematurely due to fulminant disease. All other biopsies were obtained from the right colon.

**Table 1:** Demographic and clinical characteristics of study patients.

	CD	UC	Non-IBD
	(n = 39)	(n = 18)	(n = 39)
**Male (%)**	25 (64)	11 (61)	18 (46)
**Age, years [median (IQR)]**	13.1 (10.8–15.9)	14.9 (12.7–16.2)	14.7 (11.3–16.3)
**Disease Location (Paris classification)**	L1: Ileal: 15 L2: Colonic: 4 L3: Ileocolonic: 15 L4: Upper tract: 18 (Upper tract only 4)	E1: Proctitis: 1 E2: Left-sided UC: 4 E4: Pancolitis: 13	-
**PCDAI [median (IQR)]**	35 (23–43)	-	-
**PUCAI [median (IQR)]**	-	50 (33–69)	-
**CRP (mg/L) [median (IQR)]**	26.5 (12.6–49)	6.8 (1–8.1)	5.4 (0.3–3.2)
**ESR (mm/hr) [median (IQR)]**	43 (24–60)	17.0 (8–34)	13 (7–24)
**IL-6 (pg/mL) [median (IQR)]**	9 (5.7–13.7)	4.4 (2.1–8.7)	2.0 (1.4–2.7)
**Biopsy**	n = 38	n = 18	n = 22
Affected cecum	8	-	-
Affected ascending colon	21	11	1
Affected sigmoid	1	-	-
Unaffected ascending colon	8	7	21

CD, Crohn’s disease; UC, ulcerative colitis; IBD, inflammatory bowel disease; PCDAI, Pediatric Crohn’s Disease Activity Index; PUCAI, Pediatric Ulcerative Colitis Activity Index; CRP, C-reactive protein; ESR, erythrocyte sedimentation rate; IL-6, interleukin-6; SD, standard deviation; IQR, interquartile range. There were 2 patients with IBDU who are not included in the table.

Ultimately, 39 subjects were diagnosed with CD and 18 subjects were diagnosed with UC. Despite extensive evaluation, two remained IBD unclassified (IBDU). Diagnoses of the 39 symptomatic controls included infectious colitis, juvenile polyp, and functional intestinal disorders. Patients with CD and UC had predominantly ileocolonic disease and pancolitis, respectively, consistent with the most commonly reported phenotypes in children and adolescents[19,26]. Disease severity varied widely with PCDAI scores ranging from 5 to 65, and PUCAI scores ranging from 0 to 80. In general, patients with CD had more systemic inflammation, as evidenced by higher CRP, ESR and IL-6 levels.

### Colonic gene expression in IBD

**Fig 1:**
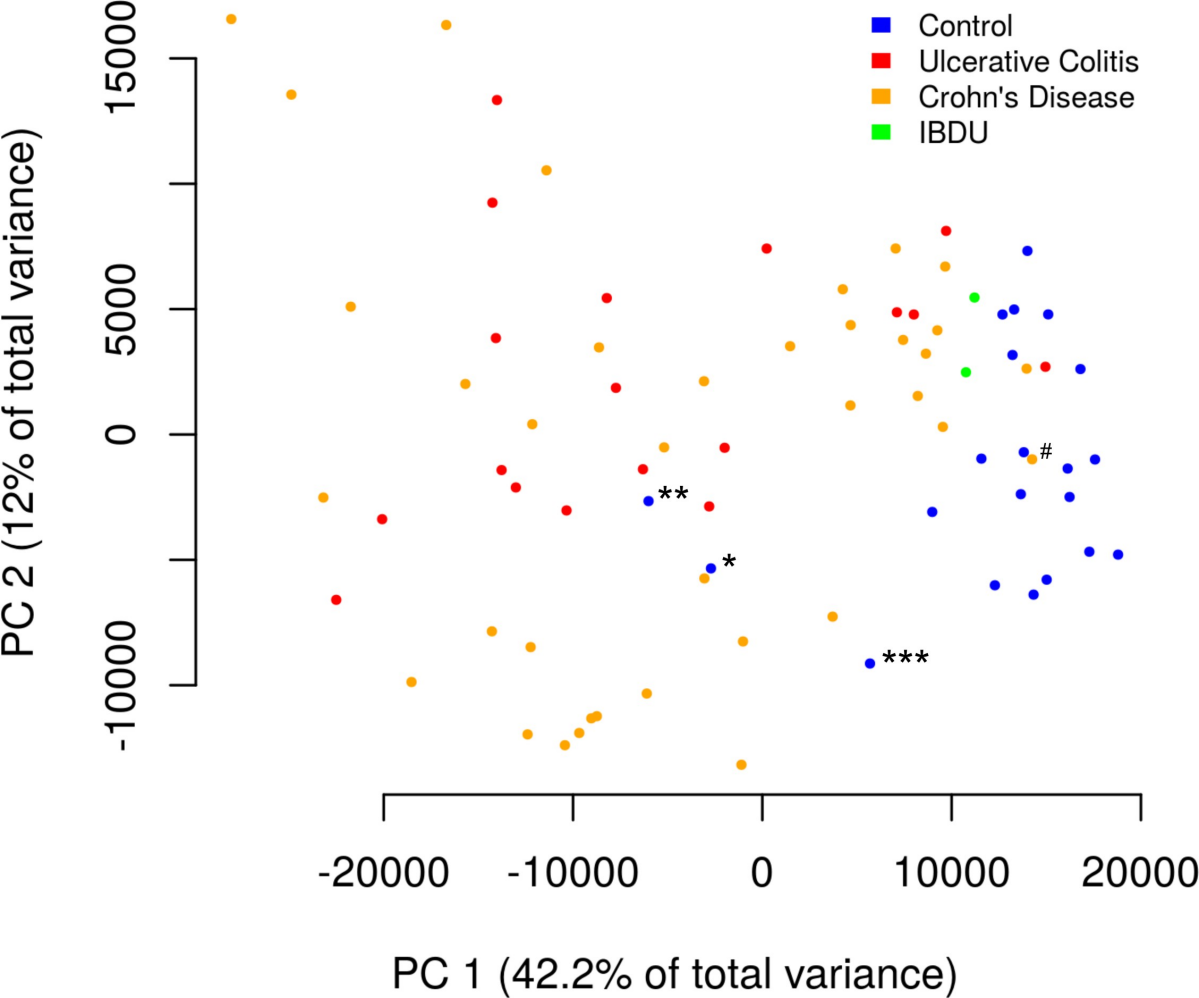
Principal components analysis of colon biopsy tissue samples from IBD and non-IBD patients. Each sample is represented as a point on the first two principal components of gene expression space, with the first principal component on the horizontal axis and the second principal component on the vertical axis. Samples from patients without IBD are shown as blue circles, while samples from patients with Crohn’s disease are shown as orange circles, samples from patients with ulcerative colitis are shown as red circles, and samples from patients with IBDU are shown as lime green circles. The first principal component mostly separates the IBD from non-IBD samples. The subject labelled * was subsequently diagnosed with Crohn’s disease and the subject labelled ** had significant rheumatologic symptoms but was lost to follow-up before a final diagnosis was made. Another subject (labelled ***) had neutrophilic esophagitis and there was loss of vascular markings in the rectosigmoid colon noted at endoscopy, but histopathologic examination and capsule endoscopy were normal. The subject labelled # presented with growth failure without intestinal symptoms.

Colonic biopsy gene expression profiles of the 58 subjects with newly diagnosed IBD were compared to those of 22 symptomatic controls. Of 18,305 annotated genes represented on the Affymetrix GeneChip^®^ Human Gene 1.0 ST array, 1,118 were significantly differentially expressed (539 upregulated, 579 downregulated) in IBD compared to symptomatic controls (S1 File). All genes were included in principal components analysis (PCA) of gene expression, an unsupervised learning procedure that identifies linear combinations of genes with coordinated expression patterns across the entire set of samples (irrespective of disease state). The first two principal components (accounting for 54.2% of total variance), showed strong separation of IBD from symptomatic controls, indicating that the disease is likely mediated by a small number of groups of genes whose expression is highly correlated (though there may be many genes in each of these few groups; Fig 1A). The relatively large first principal component also indicates that the disease-specific mRNA signature dominates all other cohort-wide transcriptional artifacts.

Notably, there were three symptomatic controls who appeared to be more similar to the IBD group. One (* in Fig 1) had isolated non-specific gastroduodenal inflammation on the index endoscopy. The subject had an appendectomy two months later and the treating clinician retrospectively applied a diagnosis of CD following review of records from another hospital. Another subject (** in Fig 1), who had eosinophilic colitis, had ongoing joint pain and was lost to follow-up prior to completion of rheumatologic evaluation. The third subject (*** in Fig 1) had neutrophilic esophagitis and there was loss of vascular markings in the rectosigmoid colon noted at endoscopy, but histopathologic examination and capsule endoscopy were normal.

### Peripheral blood gene expression in IBD

**Fig 2:**
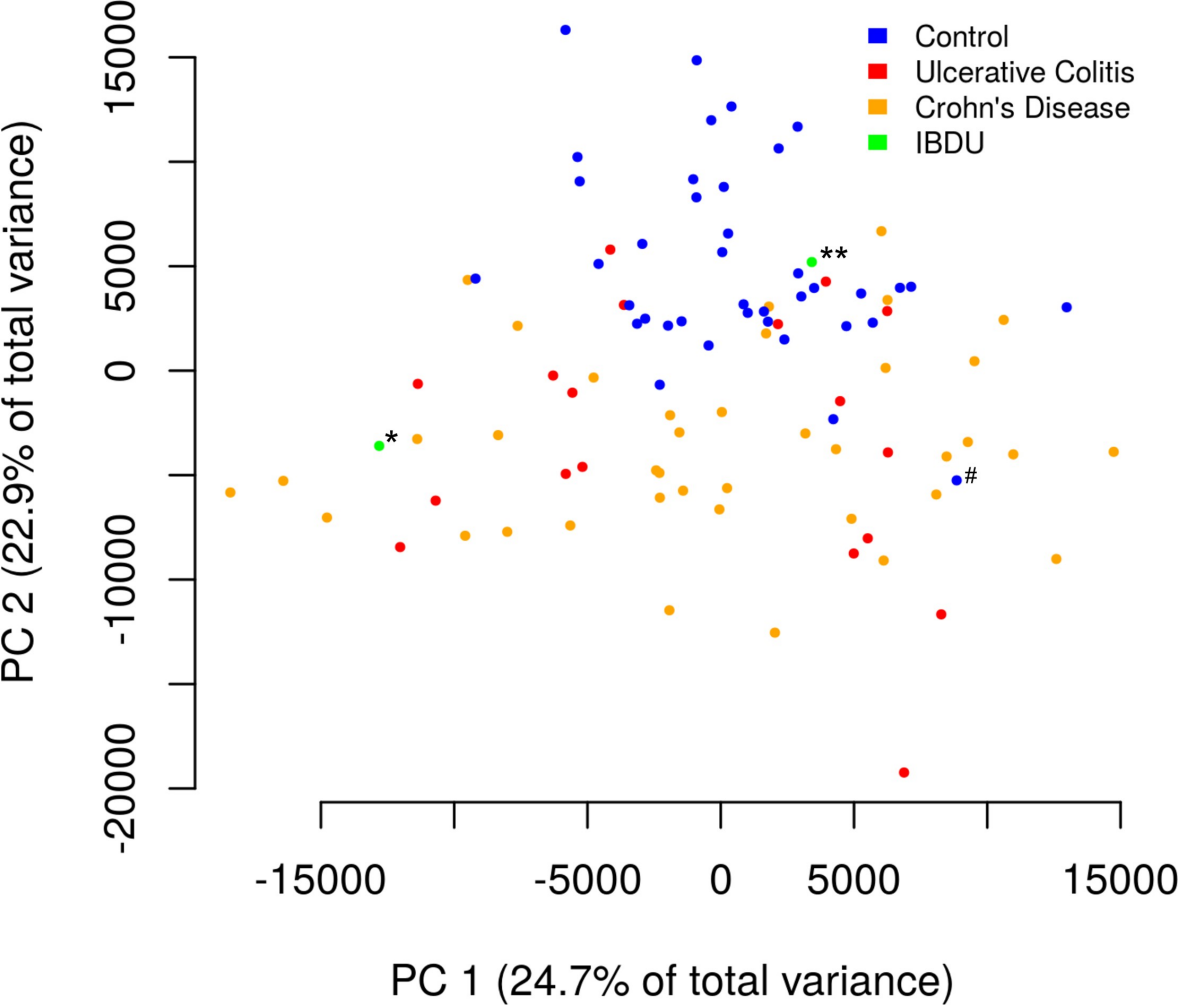
Principal components analysis of peripheral blood samples from IBD and non-IBD patients. Each sample is represented as a point on the first two principal components of gene expression space, with the first principal component on the horizontal axis and the second principal component on the vertical axis. Samples from patients without IBD are shown as blue circles, while samples from patients with Crohn’s disease are shown as orange circles, samples from patients with ulcerative colitis are shown as red circles, and samples from patients with IBDU are shown as lime green circles. The first principal component mostly separates the IBD from non-IBD samples. Please refer to text for outcomes of IBDU patients.

There were 880 genes that were significantly differentially expressed (418 upregulated, 462 downregulated) in the peripheral blood of IBD patients (n = 58) compared to symptomatic controls (n = 39; S2 File). PCA performed on all genes expressed in the peripheral blood samples revealed that, similar to colon biopsy tissue, peripheral blood demonstrates a strong IBD-specific transcriptional profile in this pediatric treatment-naïve cohort with the first two principal components representing 47.6% of total variance (Fig 2). Again, there were some patients who appeared to be more similar to those in another group, one of whom presented with joint and abdominal pain with a normal endoscopy (# in Fig 2). Of the IBDU subjects, the one who progressed to a severe form of Crohn’s disease (* in Fig 2) was much more similar to others with IBD, whereas the one that recovered without therapy (** in Fig 2) was more similar to symptomatic controls.

### Colonic biopsy and peripheral blood gene expression

Altogether, 112 genes were differentially expressed in both colon and blood (S1 and S2 Files). The direction of differential expression was concordant in 92% (62 up-regulated, 41 down-regulated). The 9 genes with discordant differential expression (*BSG*, *CDH1*, *CYP4F12*, *DSC2*, *PADI2*, *PGM1*, *RNF10*, *SRPK1*, *VDR*) were each up-regulated in blood and down-regulated in colon.

**Fig 3:**
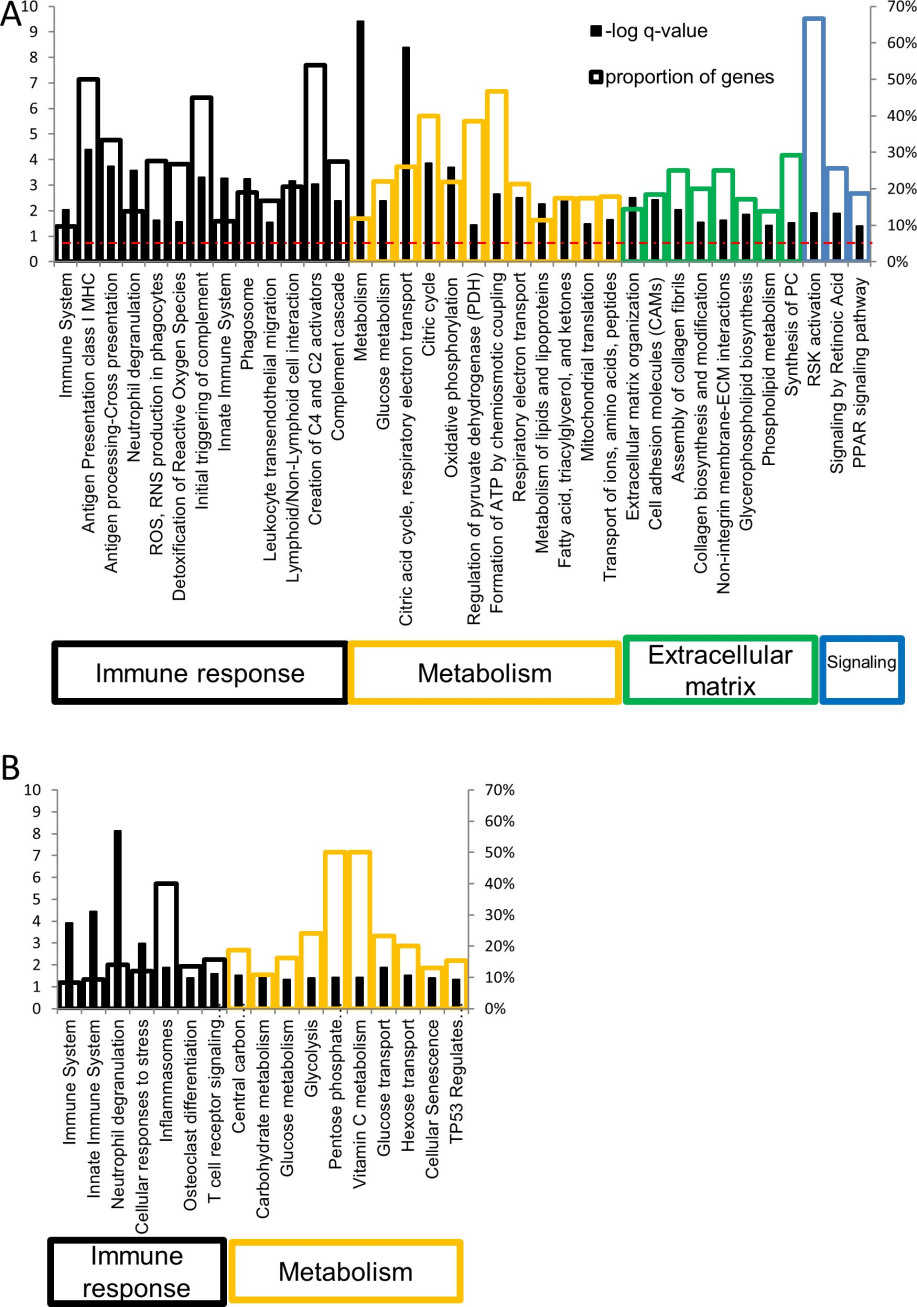
Gene sets (KEGG, Reactome) with expression levels significantly associated with IBD. (A) colon tissue. (B) peripheral blood. Solid black bars indicate the–log Q-value for each gene set (values on left axis) whereas the open colored bars indicate the proportion of genes in each set (values on right axis) whose expression levels differ significantly between those with IBD and those without IBD.

Gene Set Enrichment Analysis was performed to gain insight into the biological function of genes that were differentially expressed. After correcting for multiple hypothesis testing, the analysis identified 463 Gene Ontology (GO) sets and 67 pathways (KEGG, Reactome) that are significantly associated with the genes that were differentially expressed in colon (Fig 3A and S1 File). In addition, 282 GO sets, and 22 pathways were identified as being significantly (q-value < 0.05) associated with the genes that were differentially expressed in blood (Fig 3B and S2 File). There was on overlap of 97 GO terms and 5 pathways that were differentially overexpressed in both colon and blood. The pathways dysregulated in both tissue types were: neutrophil degranulation, immune system, innate immune system, osteoclast differentiation and glucose metabolism. This overlap was significant using the hypergeometric distribution (p<0.001). Complete lists of the gene sets and pathways can be found in S1 and S2 Files.

### Peripheral blood mRNA expression-based classification: IBD *vs*. symptomatic controls

This observed overlap between gene expression patterns in colon tissue and peripheral blood motivated us to ask whether the gene expression patterns in peripheral blood could be correlated with disease status. Therefore, we applied a Lasso regression model to the blood mRNA measurements. The predictor had non-zero coefficients for only 6 genes, *IL7R*, *UBB*, *TXNIP*, *S100A8*, *ALAS2*, and *SLC2A3* (Table 2). To establish the validity of these genes as markers of disease status, we tested the model using a publicly available independent microarray expression data set consisting of PBMC transcriptional profiles from 42 healthy adults, 59 adults with treated CD and 26 adults with treated UC. Our classifier achieved a positive predictive value (PPV) of 0.84 and negative predictive value (NPV) of 0.72 on the held-out test set. The differences in populations and chips used suggests robustness to technical measurement artifacts.

**Table 2:** Genes with non-zero coefficients in linear model of gene expression in children with active IBD compared to symptomatic controls.

Transcript	Gene name	Coefficient	Biological functions
IL7R	Interleukin 7 receptor	-0.072	B and T cell regulation; V(D)J recombination
TXNIP	Thioredoxin-interacting protein	-0.043	Thioredoxin inhibitor;NLRP3 inflammasome activation
ALAS2	5’-aminolevulinate synthase 2	0.039	Mitochondrial heme synthesis in erythrocytes
S100A8	S100 calcium binding protein A8 (a.k.a., calprotectin L1L subunit)	0.026	Innate immunity; Cell migration; Reactive oxygen species generation
UBB	Ubiquitin B	0.021	Antigen processing and presentation; Regulation of gene expression; Stress response
SLC2A3	Solute carrier family 3 member 3 (a.k.a., GLUT3)	0.12	Glucose transport into cells

In this study, comparison of gene expression between children with untreated IBD and similarly symptomatic controls revealed a distinctive molecular signature of IBD with substantial overlap of the signatures in colon biopsies and in peripheral whole blood. In both tissue types, affected gene pathways represent dysregulated immune responses and metabolic derangement that are characteristic of IBD[27]. Colon biopsies also demonstrated pathways related to cell surface receptors, extracellular matrix remodeling and localization of immune cells, which reflects tissue specific changes of inflammatory colitis. The direction of differential gene expression (up- or down-regulated) was consistent for 103 of 112 genes differentially expressed in both whole blood and colon biopsies. The 9 genes that had discordant direction of change in expression were primarily those for glycoproteins and cell-adhesion molecules, several of which have been associated with colitis (*CDH1*[28], *PADI2*[29], *VDR*[30]). Differential expression of the vitamin D receptor (VDR) in colon and peripheral blood may partially explain discordant reports regarding the role of vitamin D in IBD[31]. Thus, while gene expression signatures of IBD in colon and whole blood may not be identical, their shared structure and composition lends credence to the notion that local perturbations in gene expression are reflected in changes in gene expression in circulating blood and its constituent immune cells[32]. Furthermore, we identified six genes (*IL7R*, *UBB*, *TXNIP*, *S100A8*, *ALAS2*, and *SLC2A3*) whose expression levels in peripheral blood were predictive of disease status (Table 2). The utility of this signature was validated using an independent test in which the gene signature had 80% accuracy to distinguish adult patients receiving treatment for active IBD from healthy adults.

The demonstration of similar perturbations of gene expression in peripheral blood and in colon tissue and the nodal position of these genes in the immune signaling network supports the concept that IBD is a disease of generalized systemic immune dysregulation. Polymorphisms in IL7R, which had the largest coefficient, are associated with UC susceptibility[28]. IL7R is under balancing selective pressures, and genetic variability correlates with pathogen diversity[33]. Ubiquitin (*UBB*), one of the most conserved proteins in eukaryotic organisms, was the only gene in the regression model that was not itself significantly differentially expressed in blood; however, there were significant differences in expression of other genes involved in ubiquitin signaling (e.g., UBE2L6, USP7, PSMB8, PSME1). It is noteworthy that adheroinvasive E coli, which have been implicated in IBD pathogenesis, upregulate the ubiquitin-proteasome system via the NFκB pathway[34]. Reduced expression of *TXNIP*, an antioxidant and NFkB inhibitor which is highly expressed in lymphocytes and intestinal epithelial cells[35] in IBD patients suggests that modulation of redox signaling may be an important therapeutic target. *S100A8* encodes one subunit of calprotectin, an acute phase reactant expressed primarily by activated granulocytes that may have prognostic value[36]. Recently, peripheral blood transcript levels of the closely related homologue *S100A12* were correlated with Mayo score in adults with UC[16], which both validates our findings and further supports the potential usefulness of gene expression in peripheral blood as a marker of local disease activity. The two other genes (*ALAS2* and *SLC2A3*) whose expression levels were predictive of disease status highlight the links between the immune system and metabolism[37]. *SLC2A3* encodes GLUT3, a glucose transporter that fuels activated immune cells[38]. Intriguingly, deletion of *SLC2A3* has been identified as a protective factor in rheumatoid arthritis with an effect size that is second only to HLA genes[39]. The role of metabolic pathways in regulating immune activation has been reviewed recently[40].

Differences between colon and blood provide further insights into the pathophysiology of IBD. Transmural infiltration of neutrophils is pathognomonic for IBD, and neutrophil activation pathways were the strongest signal in peripheral blood in the current study. In the colon, the gene expression signature also reflected local events, including antigen presentation, and epithelial barrier function and repair. Expression of transmembrane transport proteins reflects both the requirement to shuttle metabolic substrates between different cellular compartments and the physiology of secretory diarrhea.

Several of the most significantly differentially expressed genes have been identified as important therapeutic targets in IBD or other autoimmune diseases, whereas others suggest promising new targets for IBD (Table 3). These include the IL12B1 receptor which is involved in signaling by IL-12 and IL-23, the cytokines targeted by ustekinumab. Our study also corroborates the finding that Lipocalin-2 (*LCN2*; also known as NGAL) expression is increased in colon tissue in IBD[41]. An antimicrobial protein that inhibits growth of iron dependent bacteria, such as E coli, Lipocalin-2 has been proposed as a fecal marker of IBD activity[42].

**Table 3:** Selected differentially expressed genes with putative mechanistic role/potential therapeutic targets in IBD.

Gene	Gene Name	Tissue location and expression change in IBD compared to symptomatic controls	Biological function	Preclinical/clinical studies
*ADM*	Adrenomedullin	Blood (↑)	Vasodilator,angiogenesis factor and anti-inflammatory	Mucosal healing in 5/7 patients with intractable UC after 14 daily infusions
*FURIN*	Furin	Blood (↑)	Proprotein convertase (substrates: IGF-1, extracellular matrix metalloproteases, TGF-β, viruses, bacterial toxins)	Copy number variants affect penetrance of CTLA4 mutations associated with early onset severe crohn’s disease.Reduced inflammation in murine collagen-induced arthritis
		Colon (↑)		
*AGTRAP*	Angiotensin II receptor type 1 associated protein	Blood (↑)	Inhibition of angiotensin II signaling	Renin-angiotensin system activation exacerbates colitis[43]. Angiotensin II type 1 receptor attenuated colitis in mice by inhibition of NFκB pathway[44]
		Colon (↑)		
*ST3GAL4; ST3GAL2*	Sialyltransferase	Blood (↑)	Glycosylation	Mucous barrier function; relationship to inflammation associated colorectal cancer; TNF induced.
		Colon (↑)		

Strengths of this study include use of paired blood and colon biopsy samples from treatment-naïve pediatric IBD patients to reduce the effects of prior treatment or lifestyle factors. Secondly, prospective study design allowed patients to be recruited prior to diagnosis using the same inclusion and exclusion criteria for IBD patients and symptomatic controls. Third, the classifier was validated in blood samples from an independent cohort of adult patients with gene expression profiling performed on a different platform[12]. The robustness of our classifier with this dataset is strong evidence that there is an underlying structure to gene expression in IBD.

A potential weakness of this study is the inclusion of diverse participants with varying clinical expression and phenotypes. More precise definition of IBD patients may facilitate identification of subgroups; however, UC and CD are similar at a gene expression level[15] and this is supported by the robustness of our classifier to identify patients with different IBD subtypes. While the use of symptomatic controls may obscure the signal of gastrointestinal symptoms, it does suggest that gene expression may be used to distinguish IBD from other causes of gastrointestinal symptoms.

## Conclusions

Treatment-naïve inflammatory bowel disease is associated with characteristic gene expression profiles in colon tissue and in peripheral blood. Overlap between the gene expression signatures in the affected tissue and peripheral whole blood suggests that IBD affects gene expression both locally in the colon and in circulating immune cells in a characteristic and reproducible manner. With this in mind, we used our gene expression data from peripheral blood to develop a linear regression model. The six transcripts identified had 80% accuracy to predict IBD status in an independent cohort of adults with and without IBD. This result was unexpected given the differences in study populations and gene chips used, which supports the robustness of our findings that gene expression profiling of peripheral whole blood reflects tissue-specific changes in IBD. Additional larger studies of well-defined cohorts are needed to determine whether patterns of RNA expression in peripheral blood may be a useful adjunct to current markers of disease activity in patients with IBD.

## Supporting information

S1 Table Real-time quantitative PCR validation of selected microarray data. (PDF)

S2 Table Sex distribution. (PDF)

S1 File Genes significantly differentially expressed in colon biopsies from IBD patients and symptomatic controls. (XLSX)

S2 File Genes significantly differentially expressed in peripheral blood from IBD patients and symptomatic controls. (XLSX)
