# Supplementary material for: Concordance between gene expression in peripheral whole blood and colonic tissue in children with inflammatory bowel disease
Source: PLoS One. 2019 Oct 16;14(10):e0222952. doi: 10.1371/journal.pone.0222952 (PMC6795427; doi:10.1371/journal.pone.0222952)
Supplement: S1 Table — (PDF) [file pone.0222952.s001.pdf]

S1 Table

Real-time Quantitative PCR Validation of Selected Microarray Data

Gene	p-value
ALDH1A2	3.71E-07
CCDC93	0.00269732
CFB	5.77E-06
CLCN6	0.041208139
CYP4X1	4.20E-07
DUOXA2	2.76E-05
KCND3	2.52E-06
LCN2	3.72E-08
MMP7	4.73E-05
PDZK1IP1	1.63E-06
PI3	5.57E-07
SAA4	7.01E-08
SLC6A14	1.62E-07
SMOC2	0.049891177
TGM2	0.000155926
TMUB2	0.008343503
VNN1	2.71E-08
ZC3H12A	0.003822516
ALDH1A2	3.71E-07
CCDC93	0.00269732
