# Supplementary material for: Concordance between gene expression in peripheral whole blood and colonic tissue in children with inflammatory bowel disease
Source: PLoS One. 2019 Oct 16;14(10):e0222952. doi: 10.1371/journal.pone.0222952 (PMC6795427; doi:10.1371/journal.pone.0222952)
Supplement: S2 Table — (PDF) [file pone.0222952.s002.pdf]

S2 Table. Sex distribution.

	Ours		GSE3365	
	Male	Female	Male	Female
UC	11	7	8	18
CD	25	14	22	38
Control	18	21	24	18
UC+CD	36	21	30	56
